# Treatment of Rapid Progression of Myopia: Topical Atropine 0.05% and MF60 Contact Lenses

**DOI:** 10.3390/vision8010003

**Published:** 2024-01-19

**Authors:** Nir Erdinest, Maya Atar-Vardi, Naomi London, David Landau, David Smadja, Eran Pras, Itay Lavy, Yair Morad

**Affiliations:** 1 Department of Ophthalmology, Hadassah-Hebrew University Medical Center, Faculty of Medicine, Hebrew University of Jerusalem, Jerusalem 9190500, Israel; nir.erdinest@mail.huji.ac.il (N.E.); dvl.eyes@gmail.com (D.L.); smadj.david@gmail.com (D.S.); itaylavy@gmail.com (I.L.); 2 The Myopia Center, Petach Tikva 4900519, Israel; yair.morad@gmail.com; 3 Department of Ophthalmology, Assaf Harofeh Medical Center, Zerifin 7033001, Israel; prase@shamir.gov.il; 4 Private Practice, 5 Even Israel, Jerusalem 9422805, Israel; imnl4u@gmail.com

**Keywords:** myopia, atropine, peripheral defocus, myopia management, myopia control, rapidly progressing myopia

## Abstract

This retrospective study evaluates the effectiveness of combining 0.05% atropine with MF60 contact lenses in managing rapid myopia progression in children over one year. The study involved three groups: the treatment group (TG) with 15 children (53% male, average age 12.9 ± 1.04), the MF group (MF) with 12 children (50% male, average age 12.8 ± 0.8) using only MF60 lenses, and the control group (CG) with 14 children (43% male, average age 12.1 ± 0.76). Baseline myopia and axial length (AL) were similar across groups, with the TG, MF, and CG showing −4.02 ± 0.70 D, −4.18 ± 0.89 D, −3.86 ± 0.99 D, and 24.72 ± 0.73 mm, 24.98 ± 0.70 mm, 24.59 ± 1.02 mm, respectively. Prior to the study, all groups exhibited significant myopia and AL progression, with no previous myopia control management. The treatment involved daily 0.05% atropine instillation, the use of MF60 lenses and increased outdoor activity. Biannual cycloplegic refraction and slit lamp evaluations confirmed no adverse reactions. After one year, the TG showed a significant reduction in myopia and AL progression (−0.43 ± 0.46 D, *p* < 0.01; 0.22 ± 0.23 mm, *p* < 0.01), whereas the CG showed minimal change (−1.30 ± 0.43 D, *p* = 0.36; 0.65 ± 0.35 mm, *p* = 0.533). The MF group also exhibited a notable decrease (−0.74 ± 0.45 D, *p* < 0.01; 0.36 ± 0.23 mm). Increased outdoor activity during the treatment year did not significantly impact myopia control, suggesting its limited additional effect in this cohort. The study concludes that the combination of 0.05% atropine and peripheral defocus soft contact lenses effectively controls myopia progression in children.

## 1. Introduction

The global prevalence and severity of myopia are on the rise, driven by a combination of genetic and environmental elements, which have been demonstrated to be interrelated [1,2,3]. The recognized risk of myopia leading to conditions like retinal detachment, macular degeneration, glaucoma, and cataracts has spurred significant research efforts to prevent its progression [2,3]. 

Treatment options include atropine, peripheral myopic defocus, orthokeratology, soft bifocal or multifocal center-distance contact lenses, and increased sunlight and bifocal or progressive addition spectacle lenses [1]. Although each treatment has shown effectiveness in particular scenarios, investigating the potential synergistic benefits of combining therapies is only beginning [4,5].

Atropine has been studied in various concentrations for its efficacy in halting myopia progression, ranging from as low as 0.01% to as high as 1%. Higher concentrations, such as 1%, have also been studied but are generally less favored due to the potential for more pronounced side effects [6,7,8,9,10]. These can include photophobia, difficulty with near vision due to pupil dilation, and the risk of systemic absorption leading to dry mouth or urinary retention. The higher the concentration, the greater the likelihood of these side effects occurring. Therefore, lower concentrations like 0.01% and 0.05% are often preferred as they balance efficacy and minimal side effects [2,3,11].

Atropine at a concentration of 0.05% is particularly effective in slowing down myopia progression in children in their first year of treatment. However, its efficacy can vary depending on the child’s age [1,2,3]. Younger children tend to respond less effectively to this concentration and may require higher doses to achieve comparable outcomes to those in older children. A study was recently published In a US-based, randomized, double-masked clinical trial involving 187 children aged 5–12 with low to moderate myopia, 0.01% atropine eye drops did not slow myopia progression or axial elongation over a 24-month treatment period and a 6-month observation period [12].

Multiple studies have corroborated the effectiveness of 0.05% atropine in mitigating myopia progression. One study found that this concentration was most effective in slowing down both myopia progression and axial elongation over one year compared to other concentrations and a placebo group [1,2,3].

Peripheral defocus and center-distance soft contact lenses have emerged as promising strategies for myopia control. The underlying concept of peripheral defocus posits that a blurred retinal image, characterized by hyperopic blur either centrally or peripherally, stimulates ocular growth. [1,2,3]. Center-distance soft contact lenses are designed to optimize retinal image quality for points on or in front of the retina, thereby reducing the hyperopic blur and/or imposing myopic defocus. Various studies have found these lenses effective in slowing myopia progression [1,2,3]. Numerous multifocal soft contact lenses and extended-depth-of-focus soft contact lenses have been developed.

These lenses typically feature ‘positive power’ to minimize hyperopic blur or induce myopic defocus [13]. Spending time outdoors has been increasingly recognized as a valuable strategy for halting myopia progression, particularly in children [14,15,16,17]. Exposure to natural light is believed to stimulate the release of dopamine in the retina, which has been shown to inhibit the elongation of the eye, a critical factor in myopia development [1,2,3]. Moreover, outdoor activities often involve focusing on distant objects, providing a natural counterbalance to the near-work tasks like reading and screen time associated with myopia onset and progression [18,19,20,21].

The present research highlights the effectiveness of a combined approach using 0.05% atropine and custom-designed peripheral defocus soft contact lenses for managing fast-progressing myopia in children over one year.

## 2. Materials and Methods

### 2.1. Ethical Principles 

The research adhered to the principles outlined in the Declaration of Helsinki. Approval for the study was granted by the Medical Center’s Institutional Review Board (IRB), and all protocols were conducted per their guidelines. Parents were informed that their children would be participating in the study. The IRB reviewed and approved the study protocol under approval number HMO 0354-21.

### 2.2. Inclusion and Exclusion Criteria

The criteria for inclusion in the study required that each eye have a cycloplegic spherical equivalent refraction (SER) of −3.00 D or higher, along with a best-corrected visual acuity of 0.3 logMAR or better. Additionally, the children participating in the study recorded myopia progression of a minimum of −1.00 D in the year leading up to the study’s commencement. An additional criterion was that one of the parents had myopia above 3.00 D.

The study excluded participants with systemic or eye-related conditions like connective tissue disorders, strabismus, or any prior atropine treatment for either myopia progression or amblyopia. Individuals with astigmatism exceeding 1.25 D or those with a history of using rigid gas-permeable contact lenses, including orthokeratology lenses, were also excluded. Children using spherical or astigmatic soft contact lenses had to discontinue lens use for two or four weeks, respectively, before the study began.

### 2.3. Study Design

A retrospective study of 15 children in the treated group, 12 in the MF60 contact lens only (MF) group, and 14 in the control group is presented here. The TG underwent daily instillation of 0.05% atropine combined with daily wear of MF60 contact lenses, and the CG used standard single-vision spectacle lenses.

Due to the significant roles that age and myopia severity play in myopia progression, we carefully examined the MF group to create a closely matched cohort to the TC and CG groups. We achieved this by selecting participants who closely matched in age and refraction, with a narrow window of +/− 6 months for age and +/− 0.75 D for refraction. Additionally, we considered parental myopia, as it can impact the genetic predisposition to myopia in their offspring when forming these groups. Consequently, we established three novel groups encompassing a total of 41 children. Among these groups, the TC, CG, and MF groups comprised 15, 14, and 12 children, respectively.

Recognition of the environmental variables that could influence the progression of myopia prompted parents to implement preventative strategies for their offspring. Different treatment alternatives were reviewed with both the parents and the children. These included using atropine eye drops at low concentrations (either 0.01% or 0.05%), either alone or in combination with progressive addition glasses, orthokeratology, and soft contact lenses designed for peripheral defocus. Each option’s possible risks and advantages were explored, and relevant scientific research was presented and clarified. Ultimately, the decision was made to use a combination of 0.05% atropine drops and soft contact lenses that induce peripheral defocus. 

These children had no previous treatment for myopia control, nor did they have any experience with contact lenses.

The children’s binocular status indicated no amblyopia, strabismus, accommodative imbalances, or oculomotor dysfunctions. Regarding the binocular test, in the TG, all patients had orthophoria at near except for three patients with 6 exophoria (XP) and one with 4XP. In the CG, all patients had orthophoria at near except for two patients who had 4XP and one patient who had 6XP. 

The children had no systemic conditions like diabetes or autoimmune diseases that could impact their ability to wear contact lenses. Additionally, none exhibited any factors that would make contact lens use inadvisable, such as dry eyes or tarsal papillae. 

The children were provided with MF60 V1 soft contact lenses featuring a +3.00 D addition, made from PolyHema 58% material by Soflex, a CooperVision Specialty Eye Care brand. These lenses, designated CDGM3, were intended for daily use and required annual replacement. The optical zone of the lenses had a 2 mm spherical center-distance region, which transitioned into an aspheric addition with +3.00 D at a 6 mm diameter. The children were advised to wear these lenses every day of the week, and parents were given instructions to clean the lenses each night using a fresh one-step hydrogen-peroxide disinfecting solution. The pupil size was measured in 36 Lux conditions using the Binocular Accommodation Auto Ref/Keratometer WAM-5500 (Shigiya Machinery Works Ltd. Hiroshima, Grand Seiko Co Ltd., WAM-5500, Hiroshima, Japan).

The lens prescription consisted of the full spherical equivalent refractions, adjusted for vertex distance as required. Objective refraction was assessed using an auto refractometer (Nidek AR-1S, Nidek Co., Aichi, Japan), and a single practitioner consistently conducted post-mydriatic subjective refraction in a uniform examination setting.

The children in both the TC group and the MF group were instructed to complete the 8-item contact lens dry eye questionnaire (CLDEQ-8) according to the questionnaire-provided guidelines. Parents assisted in filling out the questionnaire when needed, and the CLDEQ-8 scores indicated general satisfaction with wearing contact lenses [22]. 

The lenses were aligned correctly, displaying a maximum shift of 1 mm upon blinking and negligible movement during side glances. The centration of the contact lenses was examined and found to be optimal without off-center positioning. According to the contact lens manufacturer’s instructions, the subjects should be left with an overcorrection of minus 0.25 D. Parents and children received guidance on correctly inserting, removing, and handling the lenses. The children reported that the lenses felt comfortable, and their measured visual acuity (VA) was comparable to what they experienced with their glasses.

Concurrently, the pediatric participants were instructed to instill a single drop of 0.05% atropine into each eye every evening prior to retiring for sleep. This solution was formulated by the Super-Pharm Professional pharmacy chain in Petach-Tikva, Israel, and was dispensed in 10.0 mL opaque bottles (to shield from light degradation) containing 5 mL of the solution, preserved with 0.05% benzalkonium chloride. These bottles of atropine sulfate were stored for a maximum of 21 days at a temperature of 4 °C.

A follow-up visit was conducted twelve months after dispensing, including a slit lamp evaluation and cycloplegic refraction to ensure ocular health. Participants were requested to refrain from wearing contact lenses for a full day before the assessment. After administering two doses of 1% tropicamide eye drops, spaced five minutes apart, their refraction was evaluated.

A single healthcare provider conducted the measurements for spherical equivalent (SE) refraction after pupil dilation in a consistent examination room with uniform ambient lighting. Monocular distance visual acuity was assessed using a LogMAR chart. The optical biometer (OA-2000, Tomey, Nagoya, Japan) measured axial length.

### 2.4. Statistical Analysis

Statistical evaluation of demographics, time spent outdoors pre- and post-treatment, and myopia progression in the TG, MF, and CG groups after treatments was conducted using SPSS software version 25.0 (SPSS Inc., Chicago, IL, USA). One-way analysis of variance (ANOVA) tests were utilized for this analysis.

The unpaired *t*-test was employed to analyze myopia progression within the groups themselves, both before the study and one year after treatments. This statistical method was also used to analyze CLDEQ-8 data between the TG and MF groups, utilizing SPSS software. In all cases, a *p*-value below 0.05 was considered statistically significant.

## 3. Results

In the TG, 53% male, average age 12.87 ± 1.04, 12 (50% male, average age 12.8 ± 0.8), participants wore MF60 contact lenses only. In the CG, 43% of participants were male with an average age of 12.1 ± 0.76. In TG, MF and CG, the baseline average myopia and axial length (AL) were −4.02 ± 0.70 D, −4.18 ± 0.89 D and −3.86 ± 0.99 D, and 24.72 ± 0.73 mm, 24.98 ± 0.70 mm and 24.59 ± 1.02 mm, respectively (Table 1).

**Figure 1:**
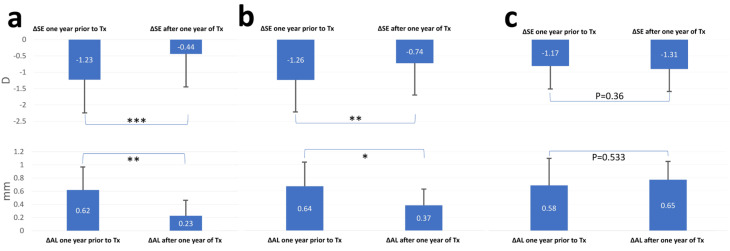
Myopia progression one year before and after treatment in the treatment group and the control group. The change in SE and AL in the TG (**a**), MF (**b**) and CG (**c**) one year before and one after starting the treatment. (**a**) One asterisks represent statistical significance (*p* < 0.05), Two asterisks represent statistical significance (*p* < 0.01), and three asterisks represent statistical significance (*p* < 0.001) for treatments. The change in SE before and after treatment showed a high statistically significant decrease. (**b**) The change in AL is also statistically significant when we compare one year before treatment and one year after. The change in AL and SE in the CG one year before and one after starting the treatment. Both parameters were not found to be statistically significant before and after treatment. Δ: delta, SE: spherical equivalent, TG: treatments group, CG: control group, Tx: treatment, AL: axial length.

Myopia progression one year before and after treatment, as well as comparisons between the TG, MF, and CG groups, can be found in Table 2. Myopia progression at the end of one year decreased from −1.22 ± 0.43 D to −0.43 ± 0.46 D (*p* < 0.0001) and 0.62 ± 0.34 mm to 0.22 ± 0.23 mm (*p* < 0.01) in the TG. In the CG, it started at −1.17 ± 0.31 D and at the end of the year −1.30 ± 0.43 D (*p* = 0.36) and from 0.57 ± 0.32 mm to 0.65 ± 0.35 mm (*p* = 0.53). For the MF group the decrease was from −1.26 ± 0.38 D to −0.74 ± 0.45 D (*p* < 0.01) and from 0.64 ± 0.36 mm to 0.36 ± 0.23 mm (*p* < 0.05) (Figure 1). The decrease of myopia progression in the TG is nearly threefold. The mean pupil diameters were observed as 4.44 ± 0.88 mm, 4.51 ± 0.69 mm, and 7.15 ± 0.70 mm in the MF, CG and TC, respectively. The comparison between MF60 and CG showed no statistically significant difference (*p* > 0.05), but a significant difference was observed between the MF and TG (*p* < 0.001), as well as between CG and TG (*p* < 0.001). 

The increase in myopia is shown in Table 2 during the year before treatment commencement.

After one year of therapy, the average change in the SE was −0.43 ± 0.46 D, 0.72 ± 0.4 D and −1.30 ± 0.43 D in TG (*p* < 0.001) MF (*p* = 0.0062) and CG (*p* = 0.36), respectively. The average change in the AL was 0.61 ± 0.34 mm in the TG before treatment and 0.57 ± 0.32 mm in the CG (*p* = 0.69). A year after, the AL change was 0.22 ± 0.23 mm in the TG and 0.65 ± 0.35 mm in the CG (*p* = 0.0005).

When comparing each group to itself, both the change in SE and AL in a year prior to and after the start of treatment was statistically significant in the TG (*p* < 0.0001 and *p* = 0.001, respectively). Contrarily, the CG was not statistically significant in those comparisons (Figure 1).

There were no changes in binocular status or visual acuity throughout the year. Examining the eye’s front surface using fluorescein, a yellow filter, and lissamine green with a red filter confirmed the absence of corneal erosions, abrasions, or conjunctival staining. Checks for papillary conjunctivitis signs were conducted on the tarsal conjunctiva, and no erythema was observed on either the tarsal or bulbar conjunctiva. The children did not experience glare or have issues with near vision. The average scores for TC and MF60 on the CLDEQ−8 scored from 0 to 37, with higher scores indicating worse symptoms, were 10.66 ± 1.52 and 11.18 ± 1.89 (*p* = 0.3963), respectively. 

During the treatment year in our study, the recorded average daily time spent outdoors increased in groups but found insignificance. In the TG group, it rose from 1.53 ± 1.04 h to 1.97 ± 1.07 h (*p* = 0.2631). In the MF group, it rose from 1.67 ± 0.88 h to 2.25 ± 0.84 h (*p* = 0.2631), whereas in the CG group, it increased from 1.42 ± 0.91 h to 2.11 ± 0.76 h (*p* = 0.0387). In addition, the comparative analysis of the hours spent outside among the three groups during the treatment period did not reveal any statistically significant differences (*p* = 0.7298).

## 4. Discussion

Myopia is a prevalent condition in children, and its progression can lead to severe vision-threatening complications. Therefore, methods to mitigate myopia progression are of significant importance. This study points to the efficacy of combining 0.05% atropine and peripheral defocus soft contact lenses to control myopia over one year. 

Atropine eye drops have been recognized as one of the most effective treatments for slowing myopia progression. Atropine acts as a reversible competitive antagonist with a non-specific affinity for all scleral muscarinic receptor subtypes, reducing fibroblast proliferation and axial elongation [23,24]. It has been shown to lessen the activity of the epidermal growth factor receptor in scleral fibroblasts and to counter the choroidal thinning induced by hyperopic retinal defocus [23,25]. Additionally, atropine stimulates the neurotransmitter dopamine release, inhibiting eye growth. It also plays a multifaceted role in biological processes, including modulating retinal signaling pathways responsive to environmental cues and intervening in the retinal pigment epithelium to help transmit regulatory signals for eye growth from the retina to the sclera [26].

Higher concentrations of atropine, like 1%, have been used and proven highly effective. However, these higher doses often lead to side effects such as photophobia and loss of accommodation [27], which, if not treated by additional techniques such as photochromatic glasses, can cause patients to be non-compliant or even drop out of treatment [23,27,28]. The use of atropine, especially at lower concentrations, has gained interest due to its efficacy in reducing myopia progression with minimal side effects [29,30]. Research indicates that a 0.05% concentration of atropine could be the ideal dosage for managing myopia progression, especially given its relatively minimal rebound effect upon discontinuation [23,29]. This appears more effective than a 0.01% concentration, particularly for patients experiencing moderate axial elongation [8,31]. The low-concentration atropine for myopia progression (LAMP) study demonstrated a significant reduction in both the rate of refractive progression and eye elongation over the one year with 0.05% atropine [2]. This study further noted efficacy in the second year [2,32]. As highlighted in a secondary analysis of the LAMP study, age can impact how individuals respond to treatment. The third and final phase examined the effects of continuing versus stopping the treatment. Continuing the treatment was more effective in all concentrations, and 0.05% remained the optimal choice over the three years [33].

Based on the findings from the three phases of the LAMP study, 0.05% atropine has emerged as the optimal concentration for controlling myopia progression in children. This concentration was found to be the most effective in slowing down both the progression of myopia and axial elongation over three years. Furthermore, when treatment was continued, the 0.05% concentration consistently outperformed the lower concentrations of 0.025% and 0.01%. Additionally, the study found that the rebound effects were clinically minor across all concentrations, suggesting that 0.05% atropine offers a balanced approach to efficacy and long-term safety.

Though the children in all age groups (ages 4–12) tolerated various low concentrations well, treatment response to low-concentration atropine tends to be less effective in younger children. Li et al. found that younger children needed a maximum concentration of 0.05% to decrease myopia progression, similar to older children with lower concentrations [2]. The children in this study correspond to an age group that exhibited a robust and dose-dependent response. 

It can be concluded, albeit with reservations due to the small sample size, that the identical results and lack of statistical significance in the CLDEQ-8 scores between the TG group and the MF suggest that the instillation of 0.05% atropine does not significantly affect or contribute to dry eye symptoms in contact lens wearers.

In a randomized, double-masked clinical trial involving myopic children aged 8 to 12 years, the MiSight 1-day contact lens was compared to a control group [34]. The results showed a 59% less unadjusted change in spherical equivalent refraction in the test group than in the control group. Additionally, the mean change in axial length was 52% less than in the control group [34]. In the same research, effectiveness did not increase in the second and third years, yet it remained more beneficial compared to the control group that received no treatment.

The bifocal lenses in nearsighted kids (BLINK) study compared identical design single-vision [35] and center-distance soft multifocal contact lenses (a + 1.50 D addition to a + 2.50 D addition) [35]. The findings indicate a specific dioptric threshold and a minimum visual field area that needs to be blurred to attain the inhibitory effect [3]. The mechanism underlying the positive influence of optical peripheral myopic defocus is not understood [36,37,38,39]. Theories suggest that minimizing the lag in accommodation [21] and potentially inhibiting the excessive stretching of Bruch’s membrane [40] could be mechanisms at play. However, the exact retinal location, the necessary surface area, and the depth of myopic defocus required for optimal effectiveness are still unclear [25,41].

This favors using a lens such as MF60, which incorporates two concentric circles of peripheral blur, thereby providing a larger retinal area of peripheral defocus and assuring some blur in both photopic and scotopic circumstances [38].

Upon reviewing the existing literature, few studies explore the use of combined therapies for managing myopia. Nucci et al. compared defocus incorporated multiple segments (DIMS) spectacles, 0.01% atropine, and a combination of both. The study concludes that both DIMS and atropine are effective in slowing myopia progression, with the most successful results seen when combining methods [42]. Many studies and a case series highlight the effectiveness of combining 0.05% atropine with custom peripheral defocus soft contact lenses in children and a clinical trial exploring the combination of atropine with orthokeratology [5,43,44,45,46,47,48,49]. However, to date, no published information exists on the potential enhanced effectiveness or any other treatments used in conjunction with 0.05% atropine.

The enhanced outcomes observed with the combination of atropine and MF60 lenses in our study can be attributed to the notable difference in pupil size between the groups. Specifically, the group using atropine with MF60 lenses exhibited a larger pupil size (7 mm compared to 4.4 mm) than the alternative group. This enlarged pupil size likely facilitated the provision of more substantial defocus to the retina, contributing to the improved results observed in myopia control [50,51].

In the context of myopia progression, the role of outdoor activities has been substantiated by several studies. A school-based program in Taiwan promoting outdoor activities demonstrated a significant reduction in myopic shift and axial elongation, with a 54% lower risk of rapid myopia progression. This protective effect was observed in nonmyopic and myopic children, suggesting that outdoor activities could be beneficial irrespective of the initial refractive status [52].

Further, a cross-sectional study conducted in Sydney (Australia) found that higher levels of outdoor activity were associated with more hyperopic refractions and lower myopia prevalence in 12-year-old students. Interestingly, students who combined low levels of near work with high levels of outdoor activity had the most hyperopic mean refraction. This study emphasizes the protective associations of increased outdoor activity against myopia, particularly when combined with lower levels of near work [53]. Moreover, an interventional study in Taiwan demonstrated that outdoor activities during class recess significantly reduced the onset of myopia and myopic shift. The study found that such activities had a prominent effect on controlling myopia shift, especially in nonmyopic children [54]. Another study found that less outdoor activity heightens myopia risk more in children with two myopic parents than in those with fewer or none, who show the lowest risk with more outdoor time [55].

It is crucial to emphasize that past myopia progression should not be regarded as a definitive predictor of future progression. Although a child may have exhibited rapid progression in the past, it does not guarantee that they will continue to do so in the future. These children should undergo a comprehensive evaluation of all relevant factors and continually monitor myopia progression to make informed decisions about management and treatment strategies if needed.

In summary, the aim was to manage myopia’s progression through two distinct treatment methods and environmental regulation. The optical component, which involves peripheral defocus contact lenses, and the biological component, represented by atropine, work synergistically. Together, they may offer greater efficacy than when used separately. The increased outdoor activity occurred in all groups, but as it was not isolated, we cannot confidently discuss its influence on myopia progression. Despite this, the absence of a decrease in myopia progression in the CG implies that increased outdoor activity might not have had an additional effect on controlling myopia in children with myopia and a moderate rate of progression.

Long-term, randomized, double-blind studies with larger participant groups could provide more definitive insights into the effectiveness of this treatment approach and whether its efficacy remains consistent or improves over time. Exploring alternative schedules for wearing contact lenses might also positively influence treatment outcomes. Further research is needed to establish when adjustments in atropine concentration are warranted for varying rates of myopia progression.

### Limitations

The main limitations of this study are the relatively small cohorts used and the nonrandomized design, which constitute additional shortcomings to this investigation. Furthermore, a slight age difference exists between the TG, MF and CG, with mean ages of 12.87 ± 1.04 and 12.80 ± 0.86 years and 12.10 ± 0.76 years, respectively. The calculated *p* value was set at 0.0334. In research, ensuring group homogeneity is a priority, particularly regarding age, given its influence on myopia management.

The other limitations of this study are related to the unique design of the peripheral defocus correction contact lens used. The results of the current study are specific to the lens design examined and do not necessarily apply to contact lenses with different designs. Therefore, the validation of the results should be considered exclusively within the context of the MF60 lens design.

## 5. Conclusions

Combining 0.05% atropine and peripheral defocus daily replacement soft contact lenses exhibited high effectiveness in controlling myopia during one year of treatment in children exhibiting a moderate progression.

## Figures and Tables

**Table 1:** Demographics and general data of the subjects. TG: treatments group, MF: MF60 contact lens only, CG: control group, Tx: treatment, AL: axial length, AVG: average, SD: standard deviation, SE: spherical equivalent, AL: axial length, M: male, Y: year, D: diopter, mm: millimeter.

	TG	CG	MF60	*p* Value
n	15	14	12	
Gender (M)	53%	43%	50.00%	0.6692
AVG(Y) and SD	12.87 ± 1.04	12.10 ± 0.76	12.80 ± 0.86	0.0511
Range (Y)	12–14.5	11–13.5	12–15	
	Average Keratometry Readings			
AVG (mm)	7.62 ± 0.16	7.57 ± 0.14	7.60 ± 0.13	0.7616
Range (mm)	7.28–7.90	7.24–7.87	7.35–7.85	
		Daily Time Outdoor (hours) Before Tx		
AVG (H)	1.53 ± 1.04	1.42 ± 0.91	1.67 ± 0.88	0.832
Range (H)	0.00–3.50	0.00–3.00	0.50–3.00	
	Visual Acuity (logMAR) Baseline			
AVG (logMAR)	0.07 ± 0.09	0.08 ± 0.08	0.09 ± 0.08	0.8426
Range (logMAR)	0.00–0.30	0.00–0.20	0.00–0.20	
		SE Baseline		
AVG (D)	−4.02 ± 0.70	−3.86 ± 0.99	−4.18 ± 0.89	0.6193
Range (D)	−6.00 to −3.00	−6.25 to −2.75	−6.00 to −3.13	
		AL Baseline		
AVG (mm)	24.72 ± 0.73	24.59 ± 1.02	24.98 ± 0.70	0.4957
Range (mm)	23.12 to 25.72	23.23 to 26.76	23.57 to 25.87	
	12 m Myopia Progression (SE) One Year Prior to Tx			
AVG (D)	−1.22 ± 0.43	−1.17 ± 0.31	−1.26 ± 0.38	0.7234
Range (D)	−2.37 to −1.00	−2.25 to −1.00	−2.25 to −1.00	
	AL Progression One Year Prior to Tx			
AVG (mm)	0.61 ± 0.34	0.57 ± 0.32	0.64 ± 0.36	0.7535
Range (mm)	0.26–1.14	0.24–1.30	0.30–1.30	

**Table 2:** Myopia progression one year before and after treatment. SE: spherical equivalent, TG: treatments group, MF: MF60 contact lens only, CG: control group, Tx: treatment, AL: axial length. NS: not significant.

	Group	After One Year of Tx	One Year Prior to Tx
SE	TG	−0.43 ± 0.46 D	−1.22 ± 0.43 D
	MF	−0.74 ± 0.45 D	−1.26 ± 0.38 D
	CG	−1.30 ± 0.43 D	−1.17 ± 0.31 D
	TG vs. MF	ns	ns
	TG vs. CG	*p* < 0.001	ns
	MF vs. CG	*p* < 0.01	ns
AL	TG	0.22 ± 0.23 mm	0.62 ± 0.34 mm
	MF	0.36 ± 0.23 mm	0.64 ± 0.36 mm
	CG	0.65 ± 0.35 mm	0.57 ± 0.32 mm
	TG vs. MF	ns	ns
	TG vs. CG	*p* < 0.001	ns
	MF vs. CG	*p* < 0.05	ns

## Data Availability

The data are available upon request from the corresponding author.
